# Gravimetric sensors operating at 1.1 GHz based on inclined *c*-axis ZnO grown on textured Al electrodes

**DOI:** 10.1038/s41598-017-01545-2

**Published:** 2017-05-02

**Authors:** Girish Rughoobur, Mario DeMiguel-Ramos, José-Miguel Escolano, Enrique Iborra, Andrew John Flewitt

**Affiliations:** 1 Electrical Engineering Division, Department of Engineering, University of Cambridge, 9 JJ Thomson Avenue, Cambridge, CB3 0FA UK; 2 GMME-CEMDATIC-ETSI de Telecomunicación, Universidad Politécnica de Madrid, 28040 Madrid, Spain

## Abstract

Shear mode solidly mounted resonators (SMRs) are fabricated using an inclined *c*-axis ZnO grown on a rough Al electrode. The roughness of the Al surface is controlled by changing the substrate temperature during the deposition process to promote the growth of inclined ZnO microcrystals. The optimum substrate temperature to obtain homogeneously inclined *c*-axis grains in ZnO films is achieved by depositing Al at 100 °C with a surface roughness ~9.2 nm, which caused an inclination angle of ~25° of the ZnO *c*-axis with respect to the surface normal. Shear mode devices with quality-factors at resonance, *Q*_r_ and effective electromechanical coupling factors, ${{\boldsymbol{k}}}_{{\bf{eff}}}^{{\bf{2}}}$, as high as 180 and 3.4% are respectively measured. Mass sensitivities, *S*_m_ of (4.9 ± 0.1) kHz · cm^2^/ng and temperature coefficient of frequency (TCF) of ~−67 ppm/K are obtained using this shear mode. The performance of the devices as viscosity sensors and biosensors is demonstrated by determining the frequency shifts of water-ethanol mixtures and detection of Rabbit immunoglobin G (IgG) whole molecule (H&L) respectively.

## Introduction

Electro-acoustic sensors resonating at high frequencies (~1–5 GHz) are promising for low-cost, label-free and high sensitivity detection of chemical and biological species in liquid media. Surface variations such as mass loading on the transducer is electrically detected by a shift in the resonance frequency, *f*_r_, to a lower value — the gravimetric principle. The higher operating frequency of thin film bulk acoustic wave (BAW) resonators compared with the well-established quartz crystal microbalance (QCM) leads to higher mass sensitivities, *S*_m_, in the order of several kHz · cm^2^/ng compared to QCM, which have *S*_m_ of several Hz · cm^2^/ng only^1, 2^. This is because *S*_m_ increases with the square of *f*_r_ according to Sauerbrey’s equation^3^. Thin film BAW resonators are categorized by the structure used for acoustic energy confinement: the free-standing film bulk acoustic resonator (free-standing FBAR), made on a free membrane with air acting as acoustic insulation and the solidly mounted resonator (SMR), which is fabricated on an acoustic reflector made of alternating high and low acoustic impedance material layers each with quarter wavelength thickness^4^. Sensors based on FBARs and SMRs are small and are fabricated by thin-film technology, which makes them compatible with integrated circuits, enabling low-cost miniature sensor systems and multiplexing^5^. FBARs and SMRs are typically operated in the thickness longitudinal (TL) mode, however this mode is severely damped in liquids as the longitudinal wave couples to the liquid. Conversely shear waves do not compress and have lower damping in liquids, thereby making the shear resonance the preferred mode of operation for in-liquid sensing^6^. Electro-acoustic resonators operating in the shear mode are therefore necessary for biosensing applications. Such devices have already been demonstrated in DNA detection^7, 8^, viscosity sensors^9^, protein binding^10^ and human IgG detection^11^.

A piezoelectric film with a *c*-axis inclined from its surface normal is the most effective method to excite the shear resonance, and this can be achieved by using seed layers with controlled roughness combined with an off-axis deposition^12, 13^. Yanagitani *et al*.^14, 15^ utilized off-axis deposition by tilting the substrate relative to the target axis to achieve 11$\bar{2}$0 oriented or zig-zag *c*-axis ZnO films, yet the limitations are the inhomogeneity over large substrates and the complex modifications of the deposition chamber. In our previous work, polycrystalline AlN seed layers with a preferred 〈103〉 orientation are used to promote the growth of such films with ZnO^16, 17^. However the AlN seed layers are electrically insulating and piezoelectrically inactive, which consequently reduce the capacitive coupling and the electric field, hence lowering the quality-factor at resonance, *Q*_r_ and effective electromechanical coupling coefficient, ${k}_{{\rm{eff}}}^{2}$. The devices fabricated using AlN seed layers had *Q*_r_ of up to 140 in air, which decreased to less than 70 in liquids^16^. A low *Q*_r_ increases the upper limit on the minimum resolvable frequency shift, Δ*f*_min_; the minimum mass (mass resolution), *m*_r_, that can be detected is therefore large. Increasing *Q*_r_ is essential to track smaller mass attachment and improve the detection limit, *m*_r_. An electrically conductive surface with a controlled roughness promoting the growth of inclined *c*-axis ZnO is necessary to reduce the parasitic losses between ZnO and the bottom electrode. Earlier works have shown that the morphology of sputtered metals such as Al can be controlled by the substrate temperature, *T*_S_ during deposition^18, 19^. At higher temperatures, more energy is supplied to the deposition particles resulting in the higher migration mobility, which in turn favors the crystallization of the film^20^. Indeed, with higher energy the deposition particles form nucleation islands that coalesce into crystals, hence promoting the formation of oriented grains and crystalline materials^21^. By varying *T*_S_ the adatom mobility on the surface and the resulting film morphology can be tuned to achieve a surface with optimum roughness necessary for growing inclined *c*-axis piezoelectric films.

In this work, the potential of using Al as a textured surface to favor the growth of inclined *c*-axis ZnO and operating as an electrode simultaneously for shear mode gravimetric biosensors is demonstrated. The objective is to eliminate a parasitic seed layer to improve the electromechanical performance of shear mode SMRs. Al electrodes sputtered at different *T*_S_ are compared and SMRs operating in the quasi-shear mode at 1.1 GHz are fabricated to determine the suitability of this method. The responses of the SMRs with temperature and mass loading in air are characterized. In addition the performance of the devices in different ethanol-water compositions is assessed. An application of the SMRs as a sensitive gravimetric biosensor in liquid is also investigated by functionalizing the surface with streptavidin to detect biotin conjugated Rabbit immunoglobin G (IgG).

## Results and Discussion

### Structure and morphology of the Al and ZnO films

**Figure 1:**
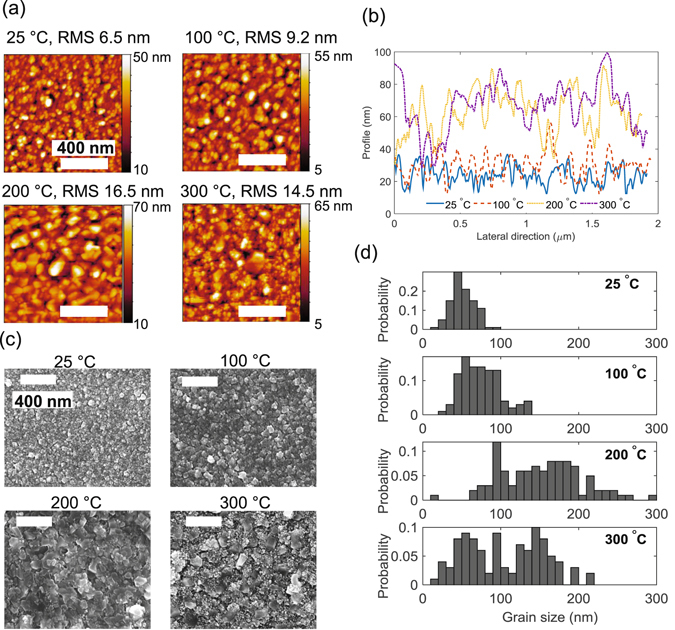
(**a**) Topography of the Al electrodes observed with AFM showing the effect of temperature on roughness, (**b**) Lateral cut profile across the AFM images showing increased roughness and grain sizes with *T*_S_ < 300 °C, (**c**) SEM images of the Al displaying the increased grain sizes as temperature increases and a mixture of large and small grains at 300 °C and (**d**) Histogram of the grain size distribution of the Al electrode at different sputtering temperatures. All scale bars in (**a**,**c**) represent 400 nm.

The film structures of the Al electrodes observed by scanning electron microscopy (SEM) and atomic force microscopy (AFM) are shown in Fig. 1.

At low temperatures (25 °C) the Al surface consists of small grains (<100 nm in size) and low roughness (<10 nm); when *T*_S_ increases, the Al grains become larger (>100 nm), and more disordered, leading to an increase in roughness (~16 nm) as shown in Fig. 1(a–c). When *T*_S_ = 300 °C, a mixture of large (>100 nm) and small Al grains are present as observed in Fig. 1(c,d). Nonetheless Al grains larger than 200 nm are less common when *T*_S_ = 300 °C compared to when *T*_S_ = 200 °C, which had grains of up to ~300 nm (Fig. 1(d)). Therefore the surface of the Al sputtered at *T*_S_ = 300 °C has lower roughness (14.2 nm) compared to when *T*_S_ = 200 °C, which has a roughness of 16.2 nm.

**Figure 2:**
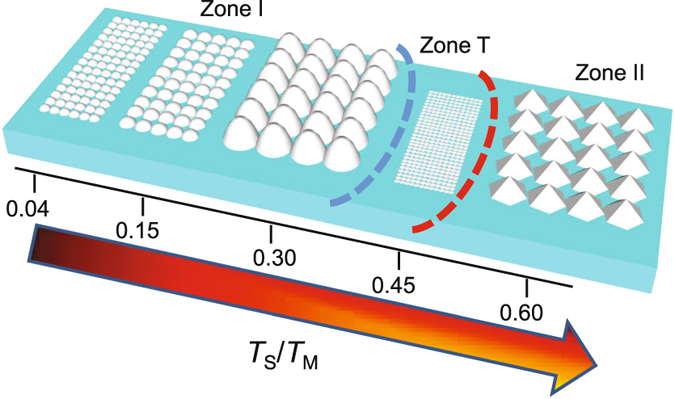
Schematic representation of a zone structure diagram for Al sputtered at constant pressure and power but different substrate temperatures showing the evolution of the grain structure with substrate temperature to melting temperature (*T*_S_/*T*_M_) ratios. In Zone I, the grains are porous and become larger as *T*_S_/*T*_M_ increases. The transition zone (T) has densely packed and smaller grains and in Zone II the film crystallizes and have large columnar grains.

The morphology of the Al films at different *T*_S_ observed in Fig. 1 can be explained by the zone structure diagram^20, 21^. An adapted schematic for the Al films sputtered at a fixed pressure and power is illustrated in Fig. 2. With a melting temperature (*T*_M_) of 660 °C the different ratios of *T*_S_/*T*_M_ can be related to the structures observed in Fig. 1(c) ^22^. When *T*_S_ is less than 200 °C, the ratio of *T*_S_/*T*_M_ is <0.3 where the film structure will be in Zone I, thus being porous and having tapered crystallites, as illustrated in Fig. 2. The roughness of these crystallites increases as *T*_S_ increases, which is observed in the growth of the Al electrodes. When *T*_S_ = 300 °C, the ratio of *T*_S_/*T*_M_ is 0.45 and the film morphology is located at the boundary of Zone T (transition) and Zone II (shown in Fig. 2) leading to a mixture of densely packed fibrous (small) grains and columnar (large) grains, as shown in the grain distribution in Fig. 1. Moreover in Zone T, the Al films have high residual compressive stress leading to poor adhesion to the substrate and hence delamination of films in contact with liquids.

**Figure 3:**
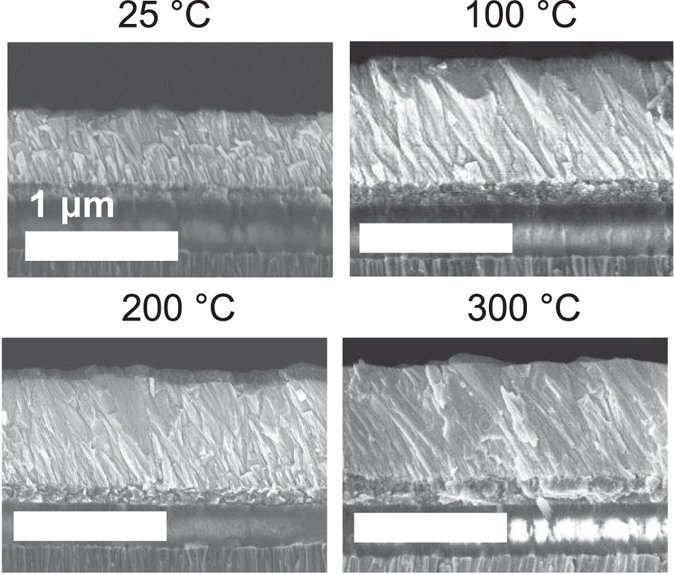
SEM cross-sections of the same magnification (25,000) of the ZnO films deposited on the Al surfaces at different *T*_S_, showing an appreciable inclination of ~30° from the surface normal. Uniform inclinations are observed at 100 °C, 200 °C and 300 °C. At 25 °C, the ZnO inclination is discontinuous as the film thickness increases. All scale bars represent 1 µm. The last two reflector layers of SiO_2_ and Mo are observable and the ZnO film for *T*_S_ is ~200 nm thinner due to rapid decrease of the deposition rate over a short distance across the samples.

SEM micrographs of the cross-section of the ZnO films subsequently deposited on the textured Al electrodes are displayed in Fig. 3. An appreciable inclination angle (~30°) from the surface normal of the ZnO film is observed in each case. However when *T*_S_ = 25 °C, the ZnO *c*-axis inclination angles are non-uniform as the ZnO microcrystals change orientation after the initial growth. This can be because of the lower roughness (6.2 nm) of the Al electrode at *T*_S_ = 25 °C, which does not favor the continuous and homogeneous growth of the inclined ZnO films. For the Al electrodes deposited at higher *T*_S_, the ZnO films have more uniform *c*-axis inclination angles as observed in Fig. 3 for *T*_S_ = 100, 200 and 300 °C. The *c*-axis of the ZnO films are inclined with the combined effect of the off-axis deposition and the surface roughness of the Al electrodes, and improved homogeneity is achieved by a uniformly rough substrate compared to a smooth surface. Yet, the position on the substrate has a significant effect on the thickness of the ZnO grown, as demonstrated in Fig. 3, where the SEM image for *T*_S_ = 25 °C shows a ZnO film, which is thinner by approximately 200 nm compared to other values of *T*_S_.

**Figure 4:**
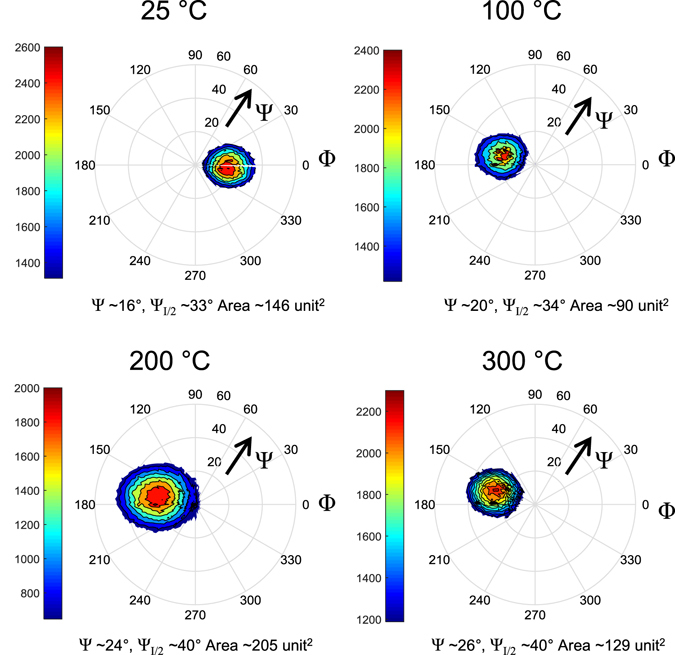
X-ray pole figures of ZnO grown on Al deposited at different *T*_S_, showing distribution of *c*-axis inclination angles with respect to the surface normal (Ψ = 0). The calculated areas enclosed within the contour surrounding the maximum intensity indicate the dispersion of the *c*-axis inclination angles. Narrower dispersions (smaller area) are observed when ZnO is deposited on Al sputtered at *T*_S_ = 100 °C. Large dispersions are observed in the case of Al sputtered at *T*_S_ of 200 °C and 300 °C. A gradual increase of the mean value of Ψ (position of center of red regions) and the FWHM (Ψ_I/2_) of the peak intensity can be observed as *T*_S_ increases from 25 °C to 300 °C.

X-ray diffraction (XRD) pole figures of the (000·2) orientation of ZnO (1 µm thick) sputtered on Al deposited at different *T*_S_ shown in Fig. 4 indicate that the *c*-axis inclination angle, Ψ, with respect to the surface normal is approximately 20°. When *T*_S_ increases from 25 °C to 200 °C, the ZnO *c*-axis inclination angles are more distributed (area of maximum contour intensity in Fig. 4 increases), and follows a similar trend to the Al grain size distribution (Fig. 1(d)) except when *T*_S_ = 100 °C. In addition the mean *c*-axis inclination angle (center of red regions in Fig. 4) of the ZnO films shifts gradually from ~20° to ~26° as the Al *T*_S_ increased from 25 °C to 300 °C. At *T*_S_ = 300 °C, the mean *c*-axis inclination angle is approximately 26° (see Supplementary Information for Φ cuts along the maximum intensities). The inhomogeneous Al grain distribution and larger grains at 200 °C and 300 °C cause a wider distribution in the Ψ value (~40°, and area enclosed in Fig. 4 are >100 unit^2^), and hence more disperse ZnO *c*-axis inclination angle around the average value as shown in Fig. 4. At 300 °C the population mix of small and large Al grains improves the intensities observed compared to when *T*_S_ = 200 °C, which is dominated by mostly large grains causing smaller intensity. The morphologies of the Al and ZnO films demonstrate that homogeneous *c*-axis inclination angles can be achieved when the Al films are sputtered at temperatures, *T*_S_ < 200 °C. In this work the optimum *T*_S_ is therefore 100 °C as the ZnO *c*-axis inclinations are more concentrated around a mean angle of 20° compared with other values of *T*_S_. At lower *T*_S_ the dispersion is large and the mean *c*-axis inclination is less than 20° as the surface does not have the optimum roughness to promote the homogeneous *c*-axis inclinations and the grains re-orient in the more energetically favorable *c*-axis oriented direction. The difference in Φ for *T*_S_ = 25 °C is caused by sample positioning in the XRD scans and has no effect on determining Ψ.

### Electro-acoustic characterization

**Figure 5:**
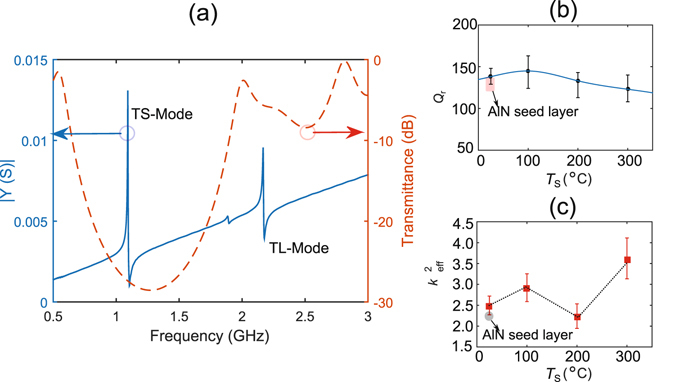
(**a**) Electrical admittance spectrum of a typical device showing a thickness shear mode resonance at 1.1 GHz and a thickness longitudinal mode resonance at 2.2 GHz. Dashed red line shows the modelled shear mode transmittance spectrum of the fabricated acoustic reflector. (**b**) Displays the average shear mode *Q*_r_ of devices measured at each Al deposition temperature; *T*_S_; the highest *Q*_r_ is achieved at 100 °C. (**c**) Shows the ${k}_{{\rm{eff}}}^{2}$ of devices, which follow a similar trend as *Q*_r_ except at 300 °C where a mean shear mode ${k}_{{\rm{eff}}}^{2}$ of 3.6% is achieved. Shaded areas in (**b**,**c**) show the performance of devices from our previous work using AlN seed layer to grow inclined *c*-axis ZnO^16^.

The spectrum of the electrical admittance, *Y*(*S*) of typical devices with *T*_S_ = 100 °C is shown in Fig. 5(a). The reflector transmittance (red dashed line in Fig. 5(a)) is simulated with the Mason transmission line model to ensure that the shear resonance is located in the main reflection lobe of the reflector, thereby achieving maximum acoustic energy confinement within the ZnO layer^23^.

The shear mode resonant frequency of the device shown in Fig. 5, *f*_S_ is 1.10 GHz while the longitudinal mode frequency, *f*_L_ is 2.18 GHz leading to a frequency ratio (*f*_L_/*f*_S_) of 1.98, which corresponds to a theoretical *c*-axis inclination angle of 33° (see Supplementary Information for equations)^24^. However this value is only an estimate as the material properties are assumed to be those of bulk ZnO. From Fig. 5(a), it can be observed that the shear resonance is located within the reflector bandwidth, thereby ensuring an efficient reflection of the shear wave. A resonance of smaller amplitude can be observed at 1.8 GHz, which corresponds to resonances from the reflector layers that are either thinner or thicker than estimated during the deposition process.

Shear mode *Q*_r_ and ${k}_{{\rm{eff}}}^{2}$ are calculated for each set of Al deposition temperatures using the standard definitions^23^

1 $${Q}_{{\rm{r}}}=\frac{{f}_{{\rm{r}}}}{2}{|\frac{{{\rm{d}}{\rm{\Phi }}}_{{\rm{Y}}}}{{\rm{d}}f}|}_{f={f}_{{\rm{r}}}}$$

2 $${k}_{{\rm{eff}}}^{2}=\frac{\pi {f}_{{\rm{s}}}}{2{f}_{{\rm{p}}}}\frac{1}{\tan (\tfrac{\pi {f}_{{\rm{s}}}}{2{f}_{{\rm{p}}}})}$$

where Φ_Y_ is the phase of the electrical admittance and *f*_s_ is the series motional resonance frequency and *f*_p_ is the lossless parallel resonance frequency. As a first approximation the frequency of maximum *Y*, hence minimum impedance, *Z*, is *f*_s_, whereas the frequency of minimum *Y*, hence maximum *Z* is *f*_p_. Using the modified Butterworth-Van-Dyke (mBVD) model, the accurate values of *f*_s_ and *f*_p_ can also be obtained (see Supplementary Information)^9, 25^.

Figure 5(b,c) are obtained by extracting *Q*_r_ and ${k}_{{\rm{eff}}}^{2}$ of 25 devices from each set and calculating the average values and standard deviation (error bars) in each direction from the mean value. SMRs with ZnO grown on Al deposited at 100 °C has the highest shear mode *Q*_r_ (155 ± 25). Therefore the optimum *T*_S_ for the electrode surface is estimated to be 100 °C. At this temperature, the Al surface has the ideal roughness to promote the homogeneous growth of the inclined ZnO, achieving a *Q*_r_ as high as 180. This value decreases at higher *T*_S_ because of the increased roughness (causing an increased series resistance of the Al electrode), which leads to additional scattering at the ZnO-Al interface resulting in parasitic losses that reduce *Q*_r_. At lower *T*_S_, the shear mode is not effectively excited leading to both a lower *Q*_r_ and ${k}_{{\rm{eff}}}^{2}$. This can probably be because the Al surface has low roughness, which is not sufficient to favor the a continuous inclined *c*-axis orientation in the ZnO grains, as shown by the lower and inhomogeneous inclination angles of ZnO when *T*_S_ = 25 °C. Despite the high ${k}_{{\rm{eff}}}^{2}$ ((3.6 ± 0.5)%) observed in Fig. 5(c) for *T*_S_ = 300 °C, the devices grown with Al at this temperature are stressed and delaminated quickly, which rendered them unsuitable for biosensing experiments. The high ${k}_{{\rm{eff}}}^{2}$ at *T*_S_ = 300 °C can be explained by the higher concentration of the ZnO inclination angles close to 30–40°, as the shear mode is most effectively excited at a *c*-axis inclination angle of ~35° ^24, 26^. Furthermore at all values of *T*_S_ for the textured Al electrodes, the values of ${k}_{{\rm{eff}}}^{2}$ both from the first approximation and from the mBVD model are higher than 2.2% (achieved with AlN seed layers^16^) because the ZnO *c*-axis inclination angles are ~25–30°, compared with AlN seed layers where inclinations of ~45° are achieved. Moreover the insulating AlN seed layers in previous works increase the parasitic losses and hence reduce the electrical to mechanical energy conversion efficiency. The *c*-axis inclination angle can be further increased by reducing the target to substrate distance to create a larger offset from the target center to the substrate. The ZnO reported in this work is actually deposited at a substrate to target distance of 17 cm. An advantage of this work is that the Al electrode and inclined *c*-axis ZnO film can be deposited in the same chamber vacuum and the process does not require any hardware modification, which is promising for large scale fabrication of electro-acoustic biosensors. In this work, the textured Al surface functions both as a seed layer and an electrode simultaneously, thus improving the electromechanical performance of the shear resonance of the SMRs. Further improvements in *Q*_r_ can potentially be achieved by the use of W due to its higher acoustic impedance compared to Al; the mechanical resonance is therefore better confined within the piezoelectric layer. However its high *T*_M_ also implies that the *T*_S_ values need to be high to achieve similar effects as with Al.

### Sensor performance

**Figure 6:**
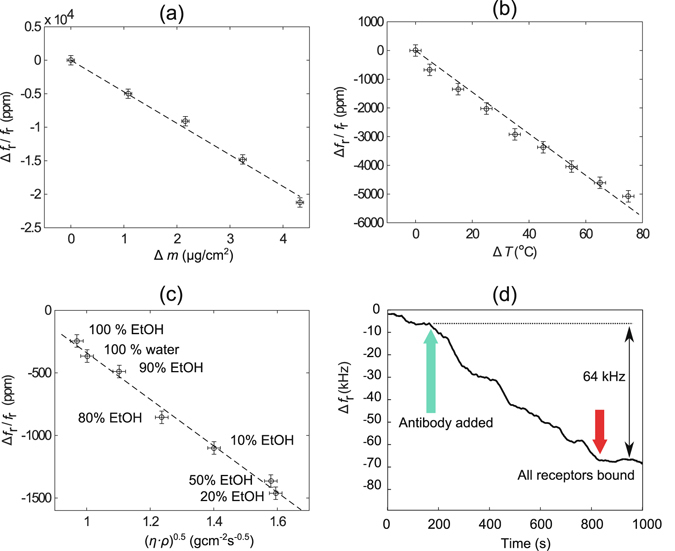
(**a**) Mass sensitivity calibration by the deposition of known thicknesses of Al on the resonator surface area, *S*_m_ = (4.95 ± 0.08) kHz · cm^2^/ng. (**b**) Temperature effect on shear mode *f*_r_, which decreases with temperature with a TCF value of −66 ppm/K. (**c**) Effect of ethanol-water mixtures with the shear mode *f*_r_. (**d**) Detection of biotin conjugated Rabbit IgG in-liquid using the shear resonance of a functionalized device, detection is complete in 600 s. All the data are for the device with Al deposited at *T*_S_ = 100 °C.

The fact that *Q*_r_ is limited to be below 180 for all the samples produced places a limit on the mass sensitivity which can be realistically achieved. Therefore in this section the SMRs with Al deposited at *T*_S_ = 100 °C are chosen to quantify the performance, due to their higher figure of merit (FOM) compared to SMRs with other *T*_S_ values. Mass sensitivity is determined by evaporating different thicknesses of Al on the active area, the mass loading of the added layer reduced the shear mode *f*_r_ linearly, as shown in Fig. 6(a).

The mass sensitivity, *S*_m_ of the shear resonance of the SMRs is calculated using equation 3:

3 $${S}_{{\rm{m}}}=-\,\frac{1}{{f}_{{\rm{r}}}}\frac{{\rm{\Delta }}{f}_{{\rm{r}}}}{{\rm{\Delta }}m}$$

where Δ*m* is the loading mass per unit area. Considering the active area, *A* = 1.42 × 10^−4^ cm^2^, and a minimum detectable noise threshold frequency of 1 kHz (${Q}_{{\rm{r}}}\approx 160$, detection limit ~1 kHz^27^), *m*_r_ is calculated as 0.20 ng/cm^2^. The devices fabricated in this work have mass sensitivities of (4.95 ± 0.08) kHz · cm^2^/ng. Figure 6(b) represents the decrease in *f*_r_ as temperature of the device increases, giving a temperature coefficient of frequency (TCF) of (−66 ± 2) ppm/K, which is close to the value (−63 ppm/K) reported elsewhere^14^. This large sensitivity to temperature should be reduced to ensure positive detection of biomolecules rather than temperature fluctuations during measurements. This can be achieved by optimizing the reflector layer materials and properties to compensate for the high intrinsic temperature sensitivity of the ZnO acoustic velocity^28^.

Water-ethanol (EtOH) mixtures having different viscosity (*η*) and density (*ρ*) cause a shift of the shear mode *f*_r_ as shown in Fig. 6(c), where a linear fit with the product (*η* · *ρ*)^0.5^ is achieved, as proposed by the Kanazawa and Gordon model^29^. This range of (*η* · *ρ*)^0.5^ is suitable for determining the viscosity sensitivity because with higher viscosity solutions, a divergence from the linear dependence is observed as the liquid cannot be assumed to be Newtonian^9^. From the measurements, a viscosity sensitivity, *S*_v_ of (−1860 ± 40) ppm/(gcm^−2^ s^−0.5^) is estimated. In water, *Q*_r_ decreased by 60%, while in EtOH, *Q*_r_ reduced by 42% (see Supplementary Information). This energy loss in the liquid corresponds to dissipation factors, *D* (1/*Q*), of 0.91% for DI water and 0.46% in EtOH. The viability of the device for in-liquid biosensing is illustrated in Fig. 6(d) as a proof of concept. After 200 s of stabilization (±5 kHz) of *f*_r_ by recirculating Binding Buffer, the biotin conjugated Rabbit IgG is added to the flow, which caused a Δ*f*_r_ of approximately −64 kHz. All streptavidin receptors are blocked after ~600 s, after which there is no further decrease in *f*_r_. This Δ*f*_r_ corresponds to a mass surface density of 1.3 ng/cm^2^ of the biotin conjugated Rabbit IgG. The binding of the biotin conjugated antibodies to the streptavidin receptors is completed in a longer time (~600 s) than reported elsewhere: the time to saturate the functionalized surface depends on the microfluidic arrangement and in this case an open system is used^30^. This experiment is carried out in a temperature controlled room to ensure that the Δ*f* is caused primarily by the mass attachment; it is also observed that the ZnO gradually reacts with the buffers, and after long immersion in liquids the surfaces are etched away. A passivation layer with either SiO_2_ or Si_3_N_4_ is therefore necessary to protect the exposed ZnO layer if such devices are to be used for in-liquid measurements.

## Conclusion

In summary textured Al electrodes are deposited by controlling the substrate temperature during deposition. As temperature increases from 25 °C to 200 °C, the Al grains become larger and the surface roughness increases. At a substrate temperature of 300 °C, a population mix of both small and large Al grains is present leading to a disperse *c*-axis inclination angles of the subsequent ZnO film. However the optimum Al surface for growing inclined *c*-axis ZnO is achieved at a substrate temperature of 100 °C with a roughness of 9.2 nm. Shear mode *Q*_r_ as high as 180 and ${k}_{{\rm{eff}}}^{2}$ of greater than 3% are achieved. The mass, temperature and viscosity sensitivities of the shear resonance are characterized. The device is functionalized and its performance as a biosensor is demonstrated by detecting biotin conjugated Rabbit IgG.

## Methods

The SMRs are fabricated by first depositing a five layer acoustic reflector stack composed of alternating layers of porous SiO_2_ and Mo. The thicknesses of the layers are optimized for a center frequency of 2.2 GHz in the longitudinal mode. Mechanical polishing with alumina slurry is used to reduce the roughness of the top SiO_2_ to less than 2 nm. Al films (~180 nm) are deposited off-axis from the target in a high target utilization sputtering (HiTUS, S500 PlasmaQuest Ltd, Hook, UK) system^31^. A power of 1100 W from a 13.56 MHz RF supply generated the remote plasma in a 0.25 Pa Ar atmosphere and the Al target (99.999% purity) is biased with a DC power of 900 W during the deposition, giving a sputtering rate of ~16 nm/min. The Al deposition process is performed at four different substrate temperatures, *T*_S_: room temperature (RT) (25 °C), 100 °C, 200 °C and 300 °C. The topography of the Al surfaces is observed with SEM (Carl Zeiss Sigma VP, Cambridge, UK) and the roughness is characterized with tapping mode AFM (Agilent 5500 scanning probe microscope, Palo Alto, CA, USA). Inclined *c*-axis ZnO films (~1 µm) are reactively sputtered off-axis at RT in the HiTUS from a 100 mm diameter 99.999% purity Zn target in a 60% O_2_ in Ar gas admixture, at a total pressure of 0.30 Pa. In this case, the launch RF power is 1250 W and a DC power of 1000 W is applied to the Zn target during the deposition. With these conditions, a sputtering rate of approximately 27 nm/min is achieved. XRD is used to determine the inclination angle of the *c*-axis of the ZnO films by measuring the XRD pole figures around the (000·2) reflection (2*θ* = 34.42°). Mo (~120 nm) is then deposited as top electrode from a 99.95% Mo target at a power of 100 W and pressure of 0.35 Pa in a DC magnetron sputtering system. Finally this top electrode is defined using standard UV photolithography followed by a dry etch process in a CF_4_/O_2_ plasma.

The electrical admittance spectra of the devices are measured in the frequency range from 0.5 to 3.0 GHz on a coplanar probe station with 150 µm pitch ground-signal-ground (G-S-G) RF probes (Picoprobes from GGB industries, USA) connected to a RF network analyzer (Keysight Technologies E5062A ENA, Santa Rosa, CA, USA). To calibrate *S*_m_, thin layers of Al (4–16 nm thick) are thermally evaporated on the resonator surface, and the corresponding *f*_r_ is extracted. The TCF of the shear resonance of the sensors is determined by tracking the *f*_r_ while heating the device on a temperature controlled Al plate. Mixtures of different EtOH and water compositions ranging from 0–100% are dropped on top of the sensor active area by means of a micropipette and Δ*f*_r_ for each composition is measured. The values for *η* and *ρ* of different water-EtOH compositions (see Supplementary Information) are obtained from Khattab *et al*.^32^.

Active areas are then patterned by standard UV photolithography and a thin layer (~50 nm) of SiO_2_ is deposited by sputtering on the detection area for the surface functionalization to assess the device performance as a biosensor. This active area is functionalized using a standard (3—Aminopropyl) triethoxysilane (APTES) - glutaraldehyde (GA) functionalization protocol^30^. First the resonator is treated with O_2_ plasma at 13.3 Pa for 2 minutes to generate OH groups on SiO_2_. In a second step, the resonator is incubated in a solution of APTES 2% (Sigma-Aldrich) in absolute EtOH for 10 minutes at RT. The surface is washed with absolute EtOH, dried with N_2_ and cured in air at 110 °C in an oven during 1 hour. Subsequently the resonator is incubated in glutaraldehyde (GA) 1% (Sigma-Aldrich) for 1 hour at RT to bind the GA to the silane. The ultimate aim of the functionalization process is to cover the entire active area of the resonator with streptavidin as receptor for the biotin marked antibody that needs to be detected. The device is incubated in streptavidin 10 g/ml (Sigma-Aldrich) in 50 mM NaCl for one hour at RT. To block the free aldehyde groups and prevent non-specific binding, the device is incubated for one hour at RT with Bovine Serum Albumin (BSA) 1% (Sigma-Aldrich) in 50 mM NaCl. Finally the device is incubated with the Binding Buffer (PBS pH 7.4, BSA 0.1%, Tween-20 0.05%) for 30 min at RT. The molecule chosen to measure the binding to the biosensor surface is Rabbit IgG whole molecule IgG (H&L) biotin conjugated (ab7074, Abcam). This antibody with concentration of 135 nM (a concentration enough to saturate the active area of the resonator) is incubated in a Binding Buffer recirculating with a peristaltic pump. A LABVIEW^®^ control is designed to automate measurements for tracking Δ*f*_r_ to monitor the attachment of the antibody to the functionalized resonator surface as a function of time. The temperature of the room is controlled and does not change by more than ±0.5 °C, which reduces the influence of temperature on the resonant frequency shifts over the measurement time (see Supplementary Information).

## Electronic supplementary material

Supplementary Information
