# Supplementary material for: Gravimetric sensors operating at 1.1 GHz based on inclined c-axis ZnO grown on textured Al electrodes
Source: Sci Rep. 2017 May 2;7:1367. doi: 10.1038/s41598-017-01545-2 (PMC5431054; doi:10.1038/s41598-017-01545-2)
Supplement: Supplementary file 1 — Supplementary Information [file 41598_2017_1545_MOESM1_ESM.pdf]

# Supplementary information: Gravimetric sensors operating at 1.1 GHz based on inclined c-axis ZnO grown on textured Al electrodes

**Girish Rughoobur[1], Mario DeMiguel-Ramos[1], José-Miguel Escolano[2], Enrique Iborra[2] and Andrew John Flewitt[1,*]**

[1]Electrical Engineering Division, Department of Engineering, University of Cambridge, 9 JJ Thomson Avenue,Cambridge, CB3 0FA, UK
[2]GMME-CEMDATIC-ETSI de Telecomunicación, Universidad Politécnica de Madrid, 28040 Madrid, Spain

*ajf@eng.cam.ac.uk

## Inclination of ZnO *c*-axis

The acoustic velocity of the longitudinal, $v^L$ and shear mode, $v^S$ are given by:

$$v^S = \left[ \frac{c_{33}^* + c_{55}^*}{2\rho} - \left( \left( \frac{c_{33}^* - c_{55}^*}{2\rho} \right)^2 + \left( \frac{c_{35}^*}{\rho} \right)^2 \right)^{0.5} \right]^{0.5}$$

$$v^L = \left[ \frac{c_{33}^* + c_{55}^*}{2\rho} + \left( \left( \frac{c_{33}^* - c_{55}^*}{2\rho} \right)^2 + \left( \frac{c_{35}^*}{\rho} \right)^2 \right)^{0.5} \right]^{0.5}$$

where $c_{33}^*$, $c_{35}^*$ and $c_{55}^*$ are the piezoelectrically stiffened elastic constants of the rotated crystal and $\rho$ is the density of ZnO and the value depends on the inclination angle, Y with respect to the surface normal, and $\rho$ is the density. The equations can be used to determine the ratio of the frequencies of the longitudinal to shear modes.

The ZnO *c*-axis inclination angle determined by XRD are shown in 3D in Fig. S1. The intensities of the peaks decrease as $T_S$ increases from 25 °C to 200 °C. At $T_S$ = 300 °C the intensity rises again as the surface roughness of the Al bottom electrode reduces to 14.2 nm.

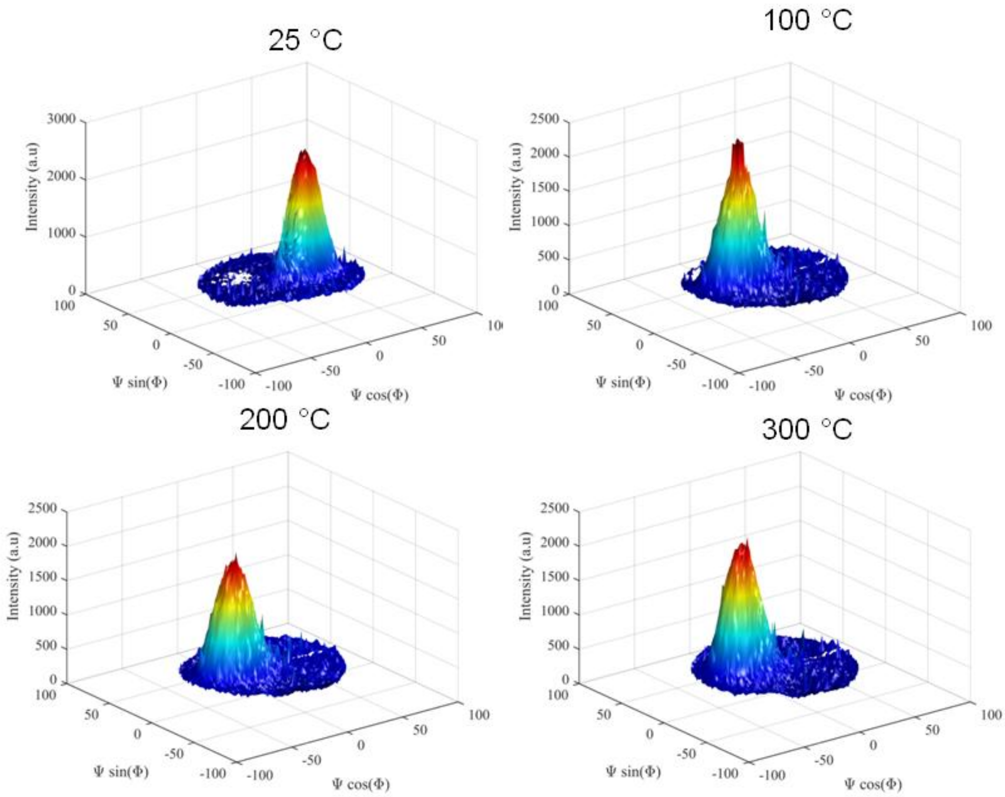


Fig. S1: XRD 3D pole figures for ZnO films deposited on Al sputtered at different $T_S$ starting from 25 °C to 300 °C. When $T_S$ = 100 °C the *c*-axis inclination angle of the ZnO has a narrower distribution than at higher temperatures.

The intensity cross-sectional plots along Φ for different $T_S$ are shown in Fig. S2 to determine the FWHM of Ψ and its approximate value.

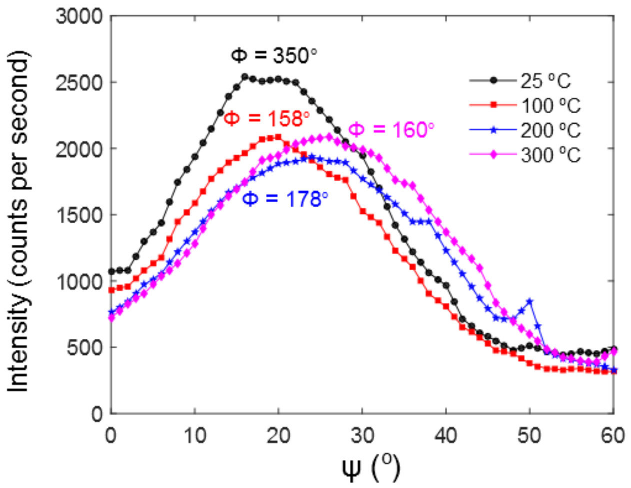


Fig. S2: XRD intensity at the position where the maximum intensities occur, with the different values of $T_S$ showing that the *c*-axis inclination angle increases as $T_S$ increases but the distribution also increases.

Quantitative data extracted from the XRD pole figures are tabulated in Table I, and demonstrate the decrease of the intensity as the surface roughness increases. In addition the grain distribution has a significant impact on the FWHM of Ψ, which increases from 33° to 40° as $T_S$ rises, thereby indicating the increased dispersion and hence inhomogeneity of the *c*-axis inclination angles.

**Table I**: Quantitative XRD data at different $T_S$ and the Ψ value at the maximum intensity.

| $T_S$ (°C) | $I_{max}$ (counts) | Ψ (°) | $\Psi_{1/2}$ (°) |
|---|---|---|---|
| 25 | 2537 | 16 | 33 |
| 100 | 2082 | 20 | 34 |
| 200 | 1933 | 24 | 40 |
| 300 | 2090 | 26 | 40 |

## Fitting with mBVD model

The resonator electrical admittance spectra are fitted with the mBVD model and the values are tabulated in Table II.

**Table II**: Quantitative XRD data at different $T_S$ and the Ψ value at the maximum intensity.

| $T_S$ (°C) | $R_m$ (Ω) | $R_s$ (Ω) | $R_0$ (Ω) | $L_m$ (μH) | $C_0$ (pF) | $C_m$ (pF) | $k^2_{eff}$ (%) | $Q_{mech}$ | FOM |
|---|---|---|---|---|---|---|---|---|---|
| 25 | 101.96 | 8.65 | 11.60 | 1.60 | 0.762 | 0.0170 | 2.7 | 108 | 2.9 |
| 100 | 64.90 | 10.22 | 13.81 | 1.45 | 0.790 | 0.0167 | 2.5 | 154 | 3.9 |
| 200 | 81.88 | 12.02 | 16.93 | 1.62 | 0.730 | 0.0154 | 2.5 | 136 | 3.4 |
| 300 | 72.33 | 8.26 | 14.12 | 1.44 | 0.731 | 0.0157 | 2.6 | 138 | 3.6 |

Using the values derived from the mBVD model in Table II, the motional series resonant frequency, $f_s$, and the lossless parallel resonant frequency, $f_p$, can be calculated as follows:[1]

$$f_s = \frac{1}{2\pi\sqrt{L_m C_m}}$$

$$f_p = \frac{\sqrt{C_0 + C_m}}{2\pi\sqrt{L_m C_0 C_m}}$$

The purely mechanical $Q$ is given by:

$$Q_{mech} = \frac{\omega_a L_m}{R_m}$$

And the figure of merit (FOM) can therefore be extracted as:[1]

$$FOM = Q \times k_{eff}^2$$

## Measurement in liquid

The electrical admittance spectra of the device without any liquid and with ethanol and water dropped on the active area are shown in Fig. S. The $Q_r$ at resonance without any load is 159, and decreases to 92 in ethanol and to 68 in water.

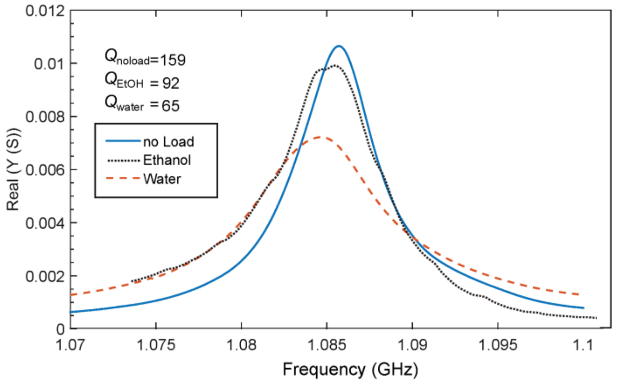


Fig. S3: Effect of water and ethanol on the thickness shear mode resonance showing a reduction in the $Q_r$ from 159 to 92 in ethanol and 65 in water. Dotted black line corresponds to EtOH, dashed red line to water and solid blue line to device without liquid.

The viscosity ($\eta$) and density ($\rho$) data for different EtOH-water compositions are obtained from Khattab *et al*.[2] The values for the compositions and for the product $(\eta \cdot \rho)^{0.5}$ are listed in the Table III.

**Table III**: Viscosity and density data for different percentages of ethanol (EtOH) in water.

| Percentage of EtOH (%) | $\rho$ (g/cm$^3$) | $\eta$ (Pa·s) | $(\eta \cdot \rho)^{0.5}$ (g·cm$^{-2}$·s$^{-0.5}$) |
|---|---|---|---|
| 0 | 1.000 | 1.003 | 1.002 |
| 10 | 0.979 | 2.004 | 1.401 |
| 20 | 0.958 | 2.655 | 1.595 |
| 50 | 0.895 | 2.787 | 1.579 |
| 80 | 0.832 | 1.837 | 1.236 |
| 90 | 0.811 | 1.500 | 1.103 |
| 100 | 0.790 | 1.190 | 0.969 |

The temperature variation of the room where the measurement is carried out and a typical frequency shift plot with time is shown in Figure S4. In addition the measurements performed to demonstrate the sensing principles last less than 30 mins, and the temperature variations are therefore not influent on the frequency shifts observed.

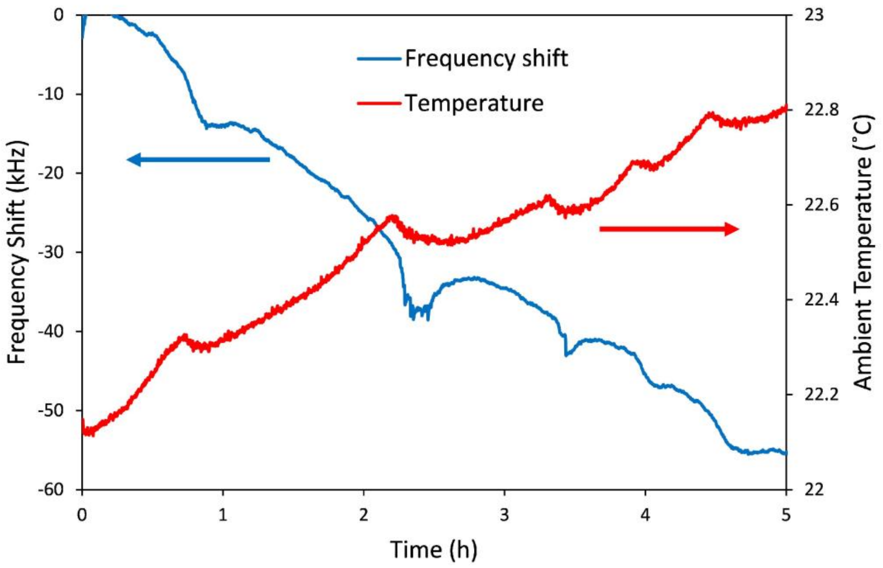


Fig. S4: Frequency shift plot and temperature variation with time of a non-functionalized resonator to illustrate that the temperature of the room changes by less than ±0.5 °C over long periods.

## References

1. Rosenbaum, J. F. Bulk Acoustic Wave Theory and Devices (Artech House, New York, 1945).

2. Khattab, I. S., Bandarkar, F., Fakhree, M. A. A. & Jouyban, A. Density, viscosity, and surface tension of water+ethanol mixtures from 293 to 323K. *Korean Journal of Chemical Engineering* **29**, 812–817 (2012).
